# Health-Related Fitness in Adults From Eight European Countries—An Analysis Based on Data From the European Fitness Badge

**DOI:** 10.3389/fphys.2020.615237

**Published:** 2021-01-08

**Authors:** Katja Klemm, Janina Krell-Roesch, Ine Lucia De Clerck, Walter Brehm, Klaus Boes

**Affiliations:** ^1^Institute of Sports and Sports Science, Karlsruhe Institute of Technology, Karlsruhe, Germany; ^2^Department of Health and Care, Artevelde University of Applied Sciences, Ghent, Belgium; ^3^Department of Sport Science, University of Bayreuth, Bayreuth, Germany

**Keywords:** Europe, motor fitness tests, physical activity, health-enhancing physical activity, health-related fitness, European Fitness Badge

## Abstract

**Background:**

There are conflicting reports about the fitness status of European adults, partly due to the lack of a standardized fitness test battery used across Europe. The European Fitness Badge (EFB) was developed in 2017 as an online-based tool to assess the health-related fitness of persons aged ≥ 18 years residing in European countries. We examined the demographic characteristics and fitness status of persons who completed the EFB between June 2017 and May 2019.

**Methods:**

We conducted a multinational study in eight European countries. Participants completed the EFB which includes 11 validated motor tests to measure endurance, strength, coordination, and flexibility performance, under the supervision of an EFB instructor in different settings (e.g., sports club sessions, public events). Two different test batteries [test profiles (TPs)] are available to distinguish between less active (TP1) and active individuals (TP2). We calculated descriptive statistics and conducted analyses of variance to examine sample characteristics and a potential impact of sex, age, body mass index (BMI), physical activity, and posture on fitness as assessed by the EFB.

**Results:**

The sample included 6,019 adults (68.7% females; mean age 52.7 years; age range 18–89 years). Participants who completed TP1 were older (TP1: 61.4 years; TP2: 44.2 years; *p* = 0.00), reported a lower level of physical activity (TP1: 3.8; TP2: 4.0; *p* = 0.00), had a higher BMI (TP1: 25.7; TP2: 24.3; *p* = 0.00) and a higher frequency of postural abnormalities (TP1: 43%; TP2: 33%; *p* = 0.00) than TP2 participants. Among 3,034 participants who completed TP2, males had higher performance in endurance, strength, and overall fitness, whereas females performed better in coordination and flexibility tests. In addition, younger age, lower BMI, and higher level of physical activity engagement were associated with better EFB test performance.

**Conclusion:**

The EFB can be used to assess the health-related fitness status of individuals aged ≥ 18 years. Our results show that TP1 and TP2 were completed by persons from the respective target groups (i.e., less active vs. active), and also confirm findings from previous studies on potential determinants of fitness such as sex or age.

## Introduction

Physical inactivity and sedentary behavior are among the main risk factors for various non-communicable diseases such as cardiovascular diseases or metabolic syndrome (Katzmarzyk, 2010; Thorp et al., 2011; Wen et al., 2011; Lee et al., 2012). These diseases are associated with high economic burden as they often lead to loss of work force or early retirement (Ding et al., 2016). Physical inactivity and sedentary behavior are highly prevalent in Europe, i.e., 46% of Europeans never engage in physical exercise or sport activities and this number has increased over the last 10 years (European Commission, 2018). One way to potentially impact people’s health-related behavior and motivate them to maintain or adopt an active lifestyle is individually tailored programs such as cardio or aerobic exercise programs. However, a prerequisite for a successful exercise program is a valid diagnosis of the current fitness status of a person (Godino et al., 2014).

We and others have reported that physical activity engagement is associated with fitness levels (Sandvik et al., 1993; Bouchard et al., 2012; Tittlbach et al., 2017). For example, in one of our previous studies based on EFB data, we reported that a higher physical activity level correlates with a higher fitness level. In addition, based on extreme value comparison, we showed that individuals with low fitness level had higher probability of being physically inactive than persons with high fitness level (Klemm et al., 2020).

Various fitness tests are used in Europe such as the German Sports Badge (Deutscher Olympischer Sportbund (DOSB), 2019), the Austrian Sport and Gymnastic Badge (Bundesministerium für Kunst et al., 2020), or the Eurofit test for adults (Oja and Tuxworth, 1995). However, studies on the fitness of adults residing in Europe mostly focused on cardiorespiratory fitness and its relationship to NCDs (Hamer and Steptoe, 2009; Ekblom-Bak et al., 2010; Zaccardi et al., 2015) or mortality (Laukkanen et al., 2001; Robsahm et al., 2016; Jensen et al., 2017; Ekblom-Bak et al., 2019). In the last 20 years, only few national studies examined the association between fitness status and potential determinants such as age or sex based on data from scientifically proven fitness tests in Europe (Ekblom et al., 2007; Crump et al., 2016). In addition, the majority was also focused on cardiorespiratory fitness. This is in line with studies from the United States, where more research regarding physical and health-related fitness has been carried out in the last decade (Blair et al., 1995; Lee et al., 1999, 2010; Hamer and Steptoe, 2009). To the knowledge of the authors, there is currently no fitness test available that comprises all health-relevant fitness dimensions, i.e., endurance, strength, flexibility, and coordination (Caspersen et al., 1985) and that can be completed by and is accessible to a broad population of adults residing in different European countries. Additionally, a recent systematic review on the decline of cardiorespiratory fitness worldwide called for a multinational surveillance system to monitor health and fitness trends (Lamoureux et al., 2019). Furthermore, research has shown that fitness tests are predominantly completed by individuals who are physically active on a regular basis and have a good fitness status (de Barreto et al., 2013; Finger et al., 2013), thereby highlighting the need of a test battery that is also appealing to individuals who are physically inactive or have a low fitness status.

To address these paucities, the EFB^1^ was developed between 2015 and 2017 and published in 2017. The EFB is a novel and innovative tool for the following reasons: (1) It is based on the internationally known HEPA concept (Martin et al., 2006); (2) it addresses the whole adult European population regardless of an individual’s physical activity level. To this end, the EFB consists of two different test profiles (TPs), i.e., one that is suitable for persons who do not engage in physical activity on a regular basis or who are older (TP1), and one for younger persons or those who regularly engage in physical activity (TP2) (Klemm et al., 2017a); (3) it includes 11 objective motor tests based on the health-oriented fitness dimensions endurance, strength, coordination, and flexibility plus additional measurements for body composition, posture, and stability (Bös et al., 2017); (4) handling and storage of data is fully automized through the EFB online data platform; (5) data are collected, i.e., the test is administered and evaluated by licensed and educated EFB instructors; (6) it was designed for exercise instructors who administer the test participants of their exercise training groups, during public events or in fitness clubs or companies; and (7) a first evaluation with regard to acceptance, feasibility, and psychometric properties of the EFB was carried out in 2016 prior to the publication of the EFB (Klemm et al., 2017b).

With regard to these strengths of the EFB, this current study has three specific aims: (1) To examine the dissemination of the EFB by summarizing the demographic characteristics (i.e., age, sex, level of physical activity, BMI, and posture) of persons who completed the EFB within the first 2 years after its inception and as stratified by country of residence. (2) To examine whether the EFB reaches the respective target groups (i.e., both less active and active persons) by calculating differences in age, level of physical activity, BMI, and posture abnormalities between participants who completed TP1 (for less active persons) vs. TP2 (for active persons; content-related validity). (3) To examine whether the data collected by the EFB are comparable to what has been reported in the literature with regard to a potential impact of sex, age, BMI, and physical activity on fitness status among participants who completed TP2.

Based on results from preliminary studies in various European countries (Finger et al., 2013; Eriksen et al., 2016; Klemm et al., 2017b) and based on the expertise of involved investigators and results from previous physical activity programs (Pahmeier et al., 2012; Bauer and Römer, 2018), we hypothesized that (1) participants who completed the EFB would reside in various European countries and would have a moderate to high level of physical activity; (2) participants who completed TP1 would be older, less physically active, and had a higher BMI than participants who completed TP2; and (3) there would be significant effect of age, sex, BMI, and physical activity engagement on fitness performance among participants who completed TP2.

## Materials and Methods

### Study Setting

The EFB is funded by the European Union (EU) (2015–2019). Sport and Gymnastic organizations as well as scientific partners from ten institutions across eight European countries were involved in the development of the EFB (Austria, Sportunion; Belgium, Artevelde University of Applied Sciences; Bulgaria, BG Be Active; Denmark, Danish Gymnastic and Sports Federation; Germany, German Gymnastic Federation, KIT; Slovenia, Sports Union Slovenia; Spain, UBAE; Europe wide, International Sports and Culture Association). Throughout the entire year, the EFB can be accessed by all interested organizations in Europe. Data are gathered online by these partner institutions. Organizations in participating countries offer completion of the EFB in different settings such as during sports club training sessions, community activities, or public events such as the European week of sports, according to their abilities and outreach. As the overarching goal is to make the EFB available to any adult residing in Europe, the only exclusion criteria of the EFB are age < 18 years and one or more items on the PAR-Q that were answered with “yes” (Bös et al., 2017). The EFB is administered to participants by licensed instructors who successfully completed a 1-day EFB instructor workshop. After completion of the EFB, each participant receives an individual certificate and additional feedback of seven pages on how to improve his/her fitness by those trained instructors. Collected data is saved on an online data platform accessible by all EFB instructors via an individualized access code. Data can be exported to Excel and SPSS through an anonymized output. The study procedures were approved by the ethics committee of the KIT (Tittlbach et al., 2017). Participation in the EFB is voluntary and all participants provided written informed consent.

### Measurements

**FIGURE 1:**
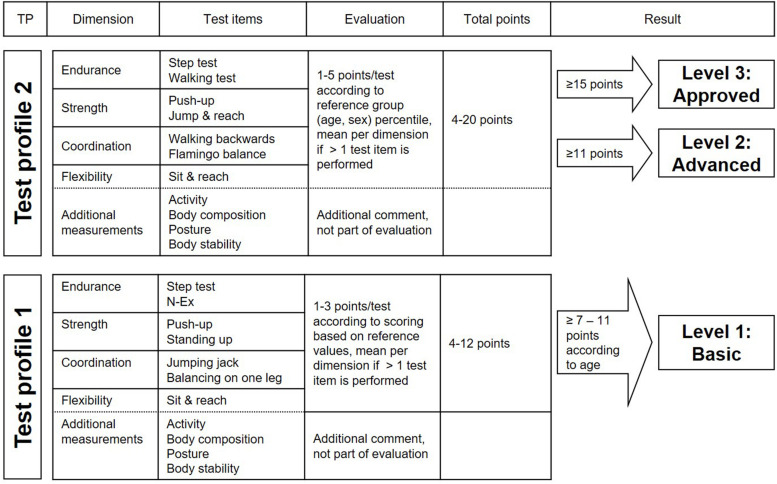
Overview of the EFB test profiles and the according evaluation.

A detailed description of the test items of the EFB has been published elsewhere (Bös et al., 2017; Klemm et al., 2017a). Briefly, the EFB has two TPs and participants are asked to choose one profile based on their level of physical activity. The level of physical activity is determined based on the N-Ex questionnaire that all participants are required to complete prior to the testing. Both TPs consist of motor performance tests to assess endurance, strength, coordination, and flexibility. Additional measurements to assess activity, posture, body height, body weight, and waist circumference are included in the EFB as well (please refer to Figure 1). Before carrying out the EFB, each participant is required to complete the PAR-Q.

Test profile 1 was designed for less active or older individuals and mainly assesses functional performance (e.g., standing up with one leg). Scores for each test range from 1 to 3 points according to the performance and based on validated reference values (Bös et al., 2017). TP1 was designed in a way that between 60 and 80% of test participants should achieve the highest score of 3 points in every test item, regardless of age and sex (exception is the sit and reach test, see Klemm et al., 2017a for details). This decision for the relatively high rates of achievement of the highest score was based on the goal of the EFB developers to motivate people who only have a low level of fitness to be physically active and thereby further improve their fitness.

The scores for each of the items are summed up based on the four dimensions of fitness, i.e., endurance, strength, coordination, and flexibility. If more than one test item is performed per dimension, the average value is calculated. In the next step, the overall test result including all four dimensions is calculated. This value ranges from 4 points (1 point in each dimension) to 12 points (3 points in each dimension). The interpretation of the total score varies by age group and is based on expert consensus (Bös et al., 2017), i.e., younger people require more points (participants aged ≤ 40 years need ≥ 11 points) than older people (participants aged > 70 years need ≥ 7 points) to reach a “basic” level. If a person does not reach the minimum number of required points, then they receive a participation certificate with feedback information.

Test profile 2 addresses physically active people and is considered performance-oriented (e.g., number of fails during balancing on a beam). The test performance is evaluated quantitatively using age- and gender-specific reference values (Bös et al., 2017) in five categories [1–5] according to percentiles. The point values refer to quintiles: 1 point = percentile rank 0–20, 5 points = percentile rank 81–100. As for TP1, results from individual tests are combined based on the motor dimensions. Overall values range from 4 points (1 point per dimension) to 20 points (5 points per dimension). Based on their test performance, participants that complete TP2 receive an “advanced” or “approved” certificate (please refer to Figure 1). If a person does not achieve a minimum of 11 points for the “advanced” level, then the person receives a participation certificate with feedback information.

To measure physical activity, the N-Ex test is included in the EFB (Jurca et al., 2005). Participants are asked to choose one of five descriptions of usual physical activities during a normal week that best reflects their activity level. The questionnaire distinguishes between “house and family care” [1], “low level activities like stair climbing” [2], “20–60 min physical activity” [3], “60–180 min physical activity” [4], and “more than 180 min physical activity” [5]. Physical activity assessed by the N-Ex reflects physical activities carried out with at least moderate intensity, i.e., with substantial increases in breathing and heart rate. When answering 1 or 2, the participants receive the recommendation of completing TP1, when answering 3, 4, or 5, it is recommended they undergo TP2.

The posture test is an observation test, i.e., the EFB instructor observes the test person during natural standing and determines the quality of posture. For the purpose of this research, we used the following two results from the posture test: no abnormality [1] and at least one abnormality [2], such as a forward head bending or a hollow lower back.

Furthermore, measurements of body weight and height were taken to calculate the BMI, and waist circumference was also assessed.

### Statistical Analysis

Participant demographics (i.e., age, sex, physical activity, BMI, posture) as stratified by country were analyzed and summarized using means (M) and SD.

In a first step, we calculated two-factorial ANOVA to examine whether there was a difference in age, level of physical activity, BMI, and posture abnormalities between participants who completed TP1 vs. TP2. We calculated main effects of TP, sex, and the interaction effect (TP × sex). Results are displayed using M, SD, and interpreted based on F-values and p-values for the main and interaction effects, and effect sizes (η^2^). Given the large sample size, it is not sufficient to only consider p-values. Rather, effect sizes may be more meaningful when interpreting the findings.

In a second step, we ran one-factorial ANOVA to examine whether performance in endurance, strength, coordination, flexibility, and overall fitness (all z-transformed variables) differed between groups of participants who completed TP2. We created the following groups: sex (males and females), age (18–39, 40–59, and >59 years), BMI (normal weight 18.5–24.9, overweight 25.0–27.4, and obese > 27.4), and physical activity level (<60, 60–180, and >180 min per week). Given the z-transformation of fitness variables, the mean for all groups is 100 (SD = 10). This allows for a comparison of performance across fitness variables and between groups. This calculation could only be done in TP2 participants due to the availability of quantitative fitness results (i.e., continuous fitness variables) in this TP.

The significance level for all analyses was set at *p* = 0.05. Partial η^2^ was categorized after Cohen (Cohen, 1992; Bauer and Römer, 2018) to low (η^2^ ≥ 0.01), medium (η^2^ ≥ 0.06), or high (η^2^ ≥ 0.14). Statistical analysis was performed using IBM SPSS Version 24.

## Results

### Demographic Breakdown of EFB Participants (Aim 1)

Table 1 provides an overview of demographic and other pertinent characteristics of participants stratified by country and EFB TP. 6,019 adults (68.7% females) aged between 18 and 89 years completed the EFB between 1 June 2017 and 31 May 2019. The mean (SD) age was 52.7 (16.7) years. The largest age group were those aged 60–69 years (27.4%), followed by participants aged 50–59 years (17.3%), aged ≥70 (15.9%), 40–49 (15.3%), 18–29 (13.1%), and 30–39 (10.9%). 73.6% of all participants reported being physically active for at least 1 h per week, 14.7% reported between 20 and 60 min of physical activity per week, and 11.7% reported no physical activity engagement in a regular week. Participants were from eight different countries, with 41% being from Denmark, 36.9% from Germany, 11.1% from Slovenia, 4.5% from Spain, 3.5% from Belgium, and 3.1% from Austria. However, due to low numbers of participants from Bulgaria (N = 1) and Czech Republic (N = 2), we did not include the results from these participants in further analyses (Table 1).

**TABLE 1:** Characteristics of study participants by country and EFB test profile.

	EFB test profile 1						EFB test profile 2					
Country (N)	N	Sex (% female)	Age (M, SD)	Activity (M, SD)	BMI (M, SD)	Posture (% no abn.)	N	Sex (% female)	Age (M, SD)	Activity (M, SD)	BMI (M, SD)	Posture (% no abn.)
**AUT** (186)	67	59.7	53.84, 13.88	3.88, 1.14	24.26, 3.20	72.6	119	58.8	40.33, 11.81	4.10, 0.91	23.65, 3.19	88.9
**BEL** (211)	18	66.7	41.89, 6.67	3.12, 1.38	24.43, 2.58	46.7	193	71.5	37.72, 8.32	3.25, 1.41	24.36, 3.00	43.5
**DEN** (2465)	1999	76.9	66.66, 6.92	3.83, 1.11	25.56, 4.33	56.2	466	57.9	44.54, 12.79	3.87, 1.06	25.37, 4.03	54.6
**ESP** (268)	166	68.7	44.12, 17.32	3.58, 1.52	27.28, 5.25	28.0	102	51.0	37.38, 12.84	4.73, 0.75	24.51, 4.02	53.9
**GER** (2220)	595	65.7	48.55, 16.18	3.82, 1.14	25.42, 4.26	75.2	1625	64.7	46.30, 16.34	4.21, 0.89	24.01, 3.61	76.4
**SVN** (666)	140	89.3	66.89, 7.12	4.16, 0.84	27.33, 4.86	22.9	526	63.5	42.03, 14.60	3.81, 1.12	24.49, 3.81	54.9
**Overall** (6019)	2985	74.3	61.37, 13.44	3.83, 1.14	25.66, 4.39	56.8	3031	63.2	44.21, 15.12	4.04, 1.04	24.32, 3.70	67.0

*Activity assessed using N-Ex and ranging from 1 to 5 with higher values reflecting higher amount of physical activity; N, number of participants; M, mean value; SD, standard deviation; BMI, body mass index; no abn., no abnormality in posture test.*

### Differences Between Test Profiles 1 and 2 (Aim 2)

The differences between TP1 and TP2 are displayed in Table 2. Overall, 49.6% of participants completed TP1 and 50.4% of participants completed TP2. Participants who completed TP1 were on average 17 years older (TP1: 61.4 years; TP2: 44.2 years; F = 1804.366, p < 0.001) and reported a slightly lower level of physical activity (TP1: 3.8; TP2: 4.0; F = 44.574, p < 0.001). There was no significant difference between males and females in either TP1 or TP2 with regard to age (F = 0.037, p = 0.847) or physical activity (F = 3.224, p = 0.073; Table 2).

**TABLE 2:** Results from two-factorial ANOVA on differences between TP1 and TP2 participants.

		N	Age (M, SD)	Activity (M, SD)	BMI (M, SD)	Posture (M, SD)
**TP1**	**f**	2,141–2,219	61.44, 13.15	3.82, 1.14	25.41, 4.47	0.41, 0.49
	**m**	735–766	61.15, 14.27	3.87, 1.16	26.59, 4.0	0.48, 0.50
	**Overall**	2,877–2,985	61.37, 13.44	3.83, 1.14	25.66, 4.39	0.43, 0.50
**TP2**	**f**	1,839–1,918	44.23, 14.58	4.02, 1.02	23.66, 3.59	0.32, 0.47
	**m**	1,022–1,116	44.09, 16.04	4.08, 1.07	25.45, 3.62	0.36, 0.48
	**Overall**	2,861–3,034	44.18, 15.13	4.04, 1.04	24.32, 3.70	0.33, 0.47
**F^*TP*^(p)**			1,804.37 (0.000)	44.57 (0.000)	151.58 (0.000)	65.85 (0.000)
**F^*sex*^(p)**			0.29 (0.589)	3.22 (0.073)	175.76 (0.000)	15.66 (0.000)
F^TP*sex^ (p)			0.04 (0.847)	0.05 (0.824)	5.62 (0.018)	1.15 (0.283)
**η^2^**			0.27	0.01	0.06	0.01

*TP1, test profile 1; TP2, test profile 2; f, females; m, males; N, number of participants; F, F-value; p, p-value; η^2^, eta square; M, mean value; SD, standard deviation. Activity assessed using N-Ex and ranging from 1 to 5 with higher values reflecting higher amount of physical activity. N ranges due to different numbers of participants in analyses for different outcome variables.*

TP1 participants also had a higher BMI (TP1: 25.7; TP2: 24.3; F = 151.579, p < 0.001) and a higher frequency of postural abnormalities (TP1: 43%; TP2: 33%; F = 65.854, p < 0.001) than TP2 participants. There was a significant sex effect on BMI (F = 175.760, p < 0.001), i.e., males had a higher BMI than females in both TP1 (males: 26.6; females: 25.4) and TP2 (males: 25.5; females: 23.7). There was also a significant TP × sex interaction effect (F = 5.620, p < 0.05), i.e., males had a 1.2 higher BMI in TP1 and a 1.8 higher BMI in TP2. Finally, there was a significant sex effect on posture abnormalities (F = 15.658, p < 0.001), i.e., males had higher frequency of postural abnormalities than females in both TP1 (males: 48%; females: 41%) and TP2 (males: 36%; females: 32%; Table 2).

### Physical Fitness by Sex, Age, BMI, and Physical Activity (Aim 3)

3,034 participants with a mean age of 44.18 years (63.2% females) completed TP2. Within this group, overall, fitness status differed significantly with regard to sex, age, BMI, and physical activity (please refer to Table 3).

**TABLE 3:** Results from one-factorial ANOVA on the impact of sex, age, body composition, and physical activity on fitness test performance.

Parameters		N	Endurance	Strength	Coordination	Flexibility	Overall fitness
**Sex**	Female (M, SD)	1,720–1,903	99.62, 9.92	96.22, 7.38	100.63, 9.19	102.39, 8.70	99.49, 9.16
	Male (M, SD)	1,004–1,109	100.66, 10.11	106.54, 10.55	98.92, 11.18	95.90, 10.72	100.87, 11.24
	F(p)		7.139 (0.008)	966.464 (0.000)	20.736 (0.000)	326.706 (0.000)	11.961 (0.001)
	η^2^		0.00	0.25	0.01	0.10	0.00
**Age**	18–39 (M, SD)	1,029–1,144	103.30, 9.22	104.94, 10.33	103.22, 6.39	101.23, 9.92	104.78, 8.48
	40–59 (M, SD)	1,274–1,384	99.52, 9.52	98.63, 8.23	100.26, 9.40	100.14, 9.42	99.29, 8.62
	>59 (M, SD)	421–485	93.35, 9.70	92.37, 7.47	91.68, 13.38	96.70, 11.03	90.46, 9.91
	F(p)		176.506 (0.000)	357.151 (0.000)	268.008 (0.000)	35.864 (0.000)	405.195 (0.000)
	η^2^		0.11	0.20	0.15	0.02	0.23
**BMI**	18.5–24.9 (M, SD)	1,732–1,872	102.35, 9.37	100.62, 9.66	101.67, 8.49	101.52, 9.73	102.24, 9.15
	25.0–27.4 (M, SD)	527–577	97.98, 9.41	100.18, 10.05	98.58, 11.25	98.81, 9.87	98.22, 9.61
	>27.4 (M, SD)	465–489	93.44, 9.54	98.13, 10.72	95.34, 11.89	96.35, 10.08	93.67, 10.38
	F(p)		185.782 (0.000)	11.970 (0.000)	89.904 (0.000)	60.007 (0.000)	162.042 (0.000)
	η^2^		0.12	0.01	0.06	0.04	0.11
**Activity**	<60 min	566–637	96.83, 9.69	98.73, 9.86	98.80, 10.73	98.39, 10.44	97.37, 9.98
	60–180 min	1,086–1,163	99.23, 10.04	98.86, 9.36	99.22, 10.61	99.96, 9.69	98.94, 9.84
	> 180 min	1,020–1,141	102.84, 9.48	101.93, 10.42	101.31, 8.91	100.99, 9.87	102.72, 9.62
	F(p)		80.859 (0.000)	33.627 (0.000)	17.748 (0.000)	14.065 (0.000)	66.217 (0.000)
	η^2^		0.06	0.02	0.01	0.01	0.05

*N, number of participants; F, F-value; p, p-value; η^2^, eta square; M, mean value; SD, standard deviation. Endurance = estimated VO_2_max based on walking or step test; strength = jump and reach test; coordination = flamingo test; flexibility = sit and reach test; N ranges due to different numbers of participants in analyses for different outcome variables. Mean and SD values are z-scores.*

Males had significantly higher performance in endurance (F = 7.139, p < 0.05), strength (F = 966.464, p < 0.001), and overall fitness (F = 11.961, p = 0.001) as compared to females. In contrast, females perform significantly better in coordination (F = 0.736, p < 0.001) and flexibility tests (F = 326.706, p < 0.001). There was also a significant age effect, i.e., participants in the youngest group (aged 18–39) performed significantly better across all tests as compared to older participants, with medium to high effect sizes (η^2^ as low as 0.11 for endurance and 0.23 for overall fitness), except for low effect size for flexibility (η^2^ = 0.02). Age as compared to sex, BMI, or physical activity also explained the highest variance in fitness performance. Furthermore, there was a significant effect of BMI on fitness status, i.e., participants with a higher BMI (≥27.4) performed significantly worse in all tests. The explained variance is low to medium with the highest effect sizes for overall fitness (η^2^ = 0.11) and endurance (η^2^ = 0.12). Finally, participants with a higher physical activity level had significantly better performance in all fitness tests and overall fitness, with effect sizes ranging between 0.01 and 0.05, except for medium effect size for endurance (η^2^ = 0.06; Table 3).

Three groups of TP2 participants, i.e., those in the youngest age group between 18–39 years, those with normal BMI of 18.5–24.9, and those with an activity level of >180 min per week achieved the best fitness results. That is, on average, their mean value was >100.00 in all four fitness dimensions and overall fitness. Overall, participants in the youngest age group had the highest level of overall fitness (M = 104.78), endurance (M = 103.30), and coordination (M = 103.22), females had the higher level of flexibility (M = 102.39), and males had the highest level of strength (M = 106.54) as compared to all other groups (Table 3).

## Discussion

Within 2 years, more than 6,000 participants residing in eight different European countries completed the EFB. With regard to our first research aim, participants who completed the EFB were, on average, physically active and middle aged. Countries such as Denmark, Germany, and Slovenia appear to be more successful in promoting the EFB as reflected by higher number of participants in these countries. One explanation might be that countries across Europe apply different strategies in promoting the EFB, e.g., integration in sport organizations, connect the EFB with other projects or cooperation with companies.

In line with our hypothesis for the second research aim, we found that participants who completed TP1 were older, less physically active, had a higher BMI and higher frequency of postural abnormalities than participants who completed TP2. This also confirms the initial differentiation of the EFB into two TPs, i.e., TP1 was designed for participants who are older and/or less physically active and may thus have a lower fitness status, and TP2 was designed for participants who are younger and/or more physically active and may thus have a higher fitness status. However, on average, almost 3/4 of all participants reported being physically active for at least 1 h per week. This indicates that even though we observed a difference between the two TPs, the EFB is mainly completed by individuals who engage in physical activity and that persons who are physically inactive may be less likely to complete the EFB. This problem of participation bias in studies on physical exercise and fitness has been described previously (de Barreto et al., 2013). More research is needed on how to reach inactive or low-active participants, and specific activity programs must be invented that address the needs of this target group (Cavill et al., 2012; Kohl et al., 2012).

In addition, with regard to the third research aim, our data show that among participants who completed TP2, age, sex, BMI, and physical activity engagement are significantly associated with fitness as assessed by the EFB. In line with our hypothesis, males had higher performance in endurance, strength, and overall fitness whereas females performed better in tests assessing coordination and flexibility. Participants in the youngest age group had significantly better results in all fitness tests than older participants. This is also in line with previous studies reporting a difference in fitness level in favor of males (Eriksen et al., 2016; Schmidt et al., 2017) and younger age groups (Ekblom et al., 2007; Milanović et al., 2013; Schmidt et al., 2017). Similarly, normal weight participants performed better than obese participants did, and participants in the higher physical activity group had a better fitness status than participants in the low physical activity group. Both findings are in line with previous research on the association between physical fitness and BMI (Farrell et al., 2002; Ross and Katzmarzyk, 2003; Tittlbach et al., 2017) as well as physical fitness and physical activity (Blair et al., 1989; Oja, 2001; Eriksen et al., 2016); albeit most of these studies focused on cardiorespiratory fitness only or did not differentiate between various motor dimensions. In addition, most of these studies were conducted in the United States.

In our study, we did not examine the efficacy of the counseling concept that has been developed and is provided to participants after completion of the EFB. As implied in the transtheoretical model (Prochaska and Velicer, 1997) and the corresponding model of health enhancing physical activity (HEPA) stages (Duan et al., 2013), motivation is an important factor for regular engagement in and maintenance of physical activity and similar health-related behaviors (Klemm et al., 2017a). Research has shown that an individually tailored counseling by an educated instructor based on a person’s fitness status is associated with desired change in health behaviors such as physical activity (Adams, 2003). In the future, we plan to examine whether the EFB can serve as a tool to enhance motivation of study participants to initiate, maintain, or enhance engaging in regular physical activity.

Our results should be interpreted in light of the strengths and weaknesses of our study. The main strengths of the EFB are its evidence-based, theoretical background, and the inclusion of validated test items that have been used by our research group for many years. This also enabled us to compare the results of EFB participants to normative values that have been developed in the past years and decades (Tittlbach et al., 2017; Klemm et al., 2020). In addition, together with the involved sports, gymnastic, or fitness organizations from eight different European countries, we were able to recruit over 6,000 participants aged ≥ 18 years who completed the EFB within 2 years after its inception. Another strength of the EFB that also distinguishes it from previously published fitness test batteries is that it is available online and provides participants with a detailed summary of test results and suggestions on how to further improve physical fitness. In addition, all data are de-identified and stored in an online database that is available for researchers per request.

However, several limitations of the study need to be noted. The major limitation of this scientific study is the selection and recruitment of study participants, the inclusion and exclusion criteria, and lacking representativeness of the study sample which has also been reported in similar previous studies (e.g., de Barreto et al., 2013). As the EFB was mainly created for the practical use within sport, gymnastic, and fitness organizations in different European countries, participants aged 18 years and older were included without a standardized recruitment strategy or stratification for criteria which could have influenced the physical activity and therefore fitness status (e.g., social background, education or living status, urban or rural regions). These limitations do not also allow for creating normative values based on the EFB test results. However, it must be noted that the EFB is deliberately designed and promoted as a fitness test that can be completed by any adult person residing in Europe. Thus, by design, we did not apply a standardized recruitment strategy but rather enabled participation of any interested person. In addition, participants completed the EFB on different occasions and in various settings and we do not have information about response rates or how response rates differed between countries. In addition, this heterogeneity is reflected by the different sample sizes in the subgroup comparisons (only one and two participants in Bulgaria and the Czech Republic compared to more than 1,000 in Denmark and Germany), and did not allow us to conduct further analyses, e.g., stratified by country of residence, by age group, or by activity level. Furthermore, we deliberately tried to be rather inclusive and the only exclusion criteria for participation in this research were an age < 18 years and one or more items that were answered with “yes” on the PAR-Q. However, the PAR-Q—albeit being a validated and often used tool in similar research studies—does not exclude conditions not related to musculoskeletal or cardiovascular limitations, although they can also influence physical activity (e.g., mental diseases). Another potential limitation of the study is seasonal changes which may impact participants’ self-reported physical activity levels, and we did not adjust analyses for this potential confounding variable. However, the fitness test battery is performed indoors and a potential impact of weather or temperature is unlikely. Finally, physical activity was assessed using the self-reported N-Ex questionnaire. Even though this is a validated questionnaire, it only provides limited information on physical activity and participants are asked to choose one of the five categories that best describes their physical activity engagement in a typical week. Future research using the EFB under controlled conditions (i.e., same instructors, similar settings, objectively measured physical activity, and representative sample, potentially through population-based randomized stratified sampling of community-dwelling persons) is thus needed to confirm our preliminary results.

Overall, to our knowledge, our study may be one of the first multinational studies that examined the impact of sex, age, BMI, and physical activity on four dimensions of health-related fitness using objective and validated motor tests in adults residing in Europe. While the EFB is a long-term initiative, data collection of the EFB is also still ongoing and in the future, more detailed and comprehensive evaluation of the physical activity and fitness status of adults in Europe based on the EFB will be possible. Furthermore, our long-term goal is to develop a European database of fitness test results based on the EFB. Taken together, results from EFB research may be used for comparisons with other regions (e.g., North America) and may also be of value to politicians and stakeholders to inform development of preventive strategies, initiatives, or plans specifically targeted to promoting physical activity and health-related fitness in Europe.

## Conclusion

The EFB is an objective tool to assess the current fitness status of individuals aged ≥ 18 years and regardless of age, sex, BMI, or physical activity level. The EFB responds to the call of many researchers for use of validated tasks that allow for an estimation of the current fitness status of a person (Watkinson et al., 2010; Godino et al., 2014; Kokkinos et al., 2017). Results from our analysis of over 6,000 adults who completed the EFB in eight European countries showed that TP1 and TP2 were completed by persons from the respective target groups (i.e., less active vs. active), and that fitness is significantly impacted by age, sex, body composition, and level of engagement in physical activity. More research is needed to confirm these preliminary findings and to also examine the potential efficacy of the EFB as a tool of motivation to initiate, maintain, or enhance engagement in regular physical activity.

## Data Availability Statement

The raw data supporting the conclusions of this article will be made available by the authors, without undue reservation.

## Ethics Statement

The studies involving human participants were reviewed and approved by Karlsruhe Institute of Technology. The patients/participants provided their written informed consent to participate in this study.

## Author Contributions

KB and WB contributed to all parts of this manuscript, particularly the introduction and discussion part. JK-R contributed to all parts of the manuscript. ID was part of the European Fitness Badge project and contributed to the methods, results, and discussion section. KK was responsible for data collection and analysis, and drafted the manuscript. All authors provided critical revision of the manuscript, and read and approved the final manuscript.

## Conflict of Interest

The authors declare that the research was conducted in the absence of any commercial or financial relationships that could be construed as a potential conflict of interest.

## Footnotes

1 www.fitness-badge.eu

## Abbreviations

ANOVA: analysis of variance
BMI: body mass index
e.g.: for example
EFB: European Fitness Badge
η ^2^: eta square
f: females
F: F-value
HEPA: health-enhancing physical activity
i.e.: is to say that
KIT: Karlsruhe Institute of Technology
M: mean value
m: males
N: number of participants
N-Ex: Non-Exercise Questionnaire
no abn.: no abnormality in posture test
P: p-value
PAR-Q: Physical Activity Readiness Questionnaire
SD: standard deviation
TP1: test profile 1
TP2: test profile 2
VO_2_max: maximal oxygen consumption
WHO: World Health Organization.
